# Quantitative determination of conjugated linoleic acid and polyunsaturated fatty acids in milk with C17:0 as internal marker – Evaluation of different methylation procedures

**DOI:** 10.1016/j.dib.2017.09.022

**Published:** 2017-09-15

**Authors:** S. Lashkari, S.K. Jensen

**Affiliations:** Department of Animal Science, Aarhus University, AU Foulum, DK-8830 Tjele, Denmark

**Keywords:** Methylation procedures, Milk fatty acid, Conjugated linoleic acid

## Abstract

Fatty acids are commonly analysed by gas chromatography as their corresponding fatty acid (FA) methyl esters (FAME). For quantitative determination of individual FA an internal standard like C17:0 is necessary. Conjugated FA and polyunsaturated fatty acid (PUFA) represents a challenge in the methylation steps, as they are sensitive to pH changes and oxidation. The present study was carried out to determine the efficiency of different methylation procedures on quantitative determination of conjugated linoleic acid (CLA), PUFA and response of internal standard. The highest response of internal standard was observed for boron trifluoride (BF_3_)/methanol and methanolic HCl followed by NaOCH_3_, while cis-9, trans-11 CLA, total CLA and PUFA was higher with methanolic HCl followed by NaOCH_3_ compared with the BF_3_ method. These data can be useful for quantitating of milk FA.

**Specifications Table**

**Table:** 

Subject area	*Analytical chemistry, food chemistry*
More specific subject area	*Milk fatty acid methylation and analysis*
Type of data	*Tables*
How data was acquired	*Gas chromatograph (Hewlett-Packard 6890 series, Agilent Technologies, Palo Alto, CA, USA) equipped with an automatic column injector (Hewlet Packard 7673), a capillary column of 30 m × 0.32 mm i.d., 0.25 μm thickness (Omegawax 320; Supelco, Sigma- Aldrich), and a flame ionization detector.*
	*The initial temperature was set at 60 °C, and then the temperature was raised to 210 °C at the rate of 2 °C/min.*
	*Fatty acids were identified by comparison of retention times with external standards (GLC-68C, Nu- Prep-Check, Elysian, MN, USA).*
Data format	*Raw data collection and analysis.*
Experimental factors	Evaluation of different methylation procedures for quantitative analyses of CLA, PUFA and C17:0 as internal standard including: alkaline catalysts, acidic catalysts and combination of alkaline and acidic catalysts.
Experimental features	*Obtained data were analyzed in a completely randomized design*
Data source location	*Aarhus university, AU Foulum, DK-8830 Tjele, Denmark*
Data accessibility	*Data are presented in this article*

**Value of the data**

• Despite all various methods employed for milk FA analyses, there are still conflicting opinions about the best method for overcoming all the difficulties posed by the analysis of complex mixtures including CLA isomers and PUFA.
• For quantitative analysis of FA, addition of internal standard is necessary. Furthermore, when an internal standard is used, the method has the capability to determine both total FA distribution and the amount of individual FA in a given sample simultaneously.
• Due to the heterogeneity of milk fatty acid, it is necessary to study the methylation procedure in order to obtain accurate quantitative and qualitative results.

## 1 Data

The internal standard response was checked to verify the effect of different methylation procedures on methylation of internal standard. Table 1 shows area under the chromatogram peak of C17:0 analyzed following the different methylation methods. The area under the chromatogram peaks was 2706 and 1834 units for the C17:0 methylated with BF_3_/methanol and methanolic HCl followed by NaOCH_3_ procedures, respectively. However, response of internal standard for the other methods was near to zero. In the present study, methanolic HCl followed by NaOCH_3_ and BF_3_/methanol methods catalyzed methylation of C17:0; therefore, analysis of milk samples with these two methods was included in the paper. Table 2 shows milk FA amount and composition following the two methylation procedures. The amount of total FA for BF_3_/methanol method was 95% of the amount detected with the methanolic HCl followed by NaOCH_3_ method. Cis-9, trans-11 CLA was 29% higher with methanolic HCl followed by NaOCH_3_ than the BF_3_/methanol method (Table 3). In addition, with methanolic HCl followed by NaOCH_3_, amount of trans-10, cis-12 CLA was 23% higher than with the BF_3_/methanol method.

**Table 1:** Area under the chromatogram peak of C17:0 analyzed following the different methylation methods.

Methods	Amounts	Percentage of BF_3_/methanol method
BF_3_/methanol	2706	100
Methanolic HCl followed by NaOCH_3_ 0.5 N	1834	67.77
NaOCH_3_ (0.5 N)	17.5	0.64
NaOCH_3_ (1 N)	16.6	0.61
Tetramethylguanidine	20.6	0.76
NaOCH_3_ 0.5 N followed by BF_3_	16.0	0.59
NaOCH_3_ 1 N followed by BF_3_	16.2	0.59
Tetramethylguanidine followed by BF_3_	18.7	0.69
NaOCH_3_ 0.5 N followed by methanolic HCl	15.1	0.55
NaOCH_3_ 1 N followed by methanolic HCl	12.6	0.46
Tetramethylguanidine followed by methanolic HCl	19.2	0.71

Each value represents the mean of the 4 blank samples.

**Table 2:** Comparison of transesterification procedures of milk fatty acid composition.

Fatty acids		Amount of fatty acid (mg kg^-1^ milk)					Amount of fatty acid (g 100 g^-1^ fatty acid)			
	BF_3_/methanol		Methanolic HCl followed by NaOCH_3_ 0.5 N	SEM	*p*-value	BF_3_/methanol		Methanolic HCl followed by NaOCH_3_ 0.5 N	SEM	*p*-value
C4-13:0^a^	0.33		0.35	0.03	0.85	12.3		12.4	0.96	0.95
C14-18:0^b^	1.35		1.40	0.011	0.86	52.7		52.2	1.78	0.90
C20-24:0^c^	0.004		0.005	0.0005	0.14	0.16		0.17	0.01	0.69
Trans vaccenic acid	99.5		85.3	16	0.76	5.8		4.3	1.71	0.68
Cis-9, trans-11 CLA^d^	8.7		12.4	0.75	0.07	0.38		0.48	0.02	0.05
Trans-10, cis -12 CLA	0.33		0.43	0.03	0.30	0.01		0.02	0.001	0.20
Total CLA^e^	9.1		12.8	0.05	0.07	0.39		0.50	0.02	0.05
n-9 MUFA^f^	0.47		0.51	0.03	0.68	18.8		21.4	1.13	0.86
n-6 PUFA^g^	0.082		0.091	0.003	0.19	3.7		3.8	0.30	0.86
n-3 PUFA^h^	0.020		0.026	0.0009	0.04	0.82		0.97	0.07	0.19
SFA^i^	1.68		1.75	0.14	0.86	65.1		64.8	2.63	0.95
MSFA^j^	0.69		0.72	0.01	0.48	30.6		29.6	2.35	0.83
UFA^k^	0.123		0.14	0.004	0.12	5.6		5.9	0.39	0.73
Total fatty acids	2482		2609	164	0.78	–		–	–	–

Each value represents the mean of the milk from six cows. SEM, Standard error of means.

a C4-13:0; ∑C2+ C4+ C6+ C8+ C10+ C12+ C13.

b C14-18:0; ∑C14+ C15+ C16+ C18.

c C20-24:0; ∑C20+C22+ C24. ^d^ CLA; conjugated linoleic acid.

e Total CLA; ∑ cis-9, trans-11 conjugated linoleic acid+ trans-10, cis-12 conjugated linoleic acid.

f n-9 MUFA (monosaturated fatty acid); ∑C16:1 n9+ C18:1 n9+ C20:1 n9+ C22:1 n9.

g n-6 PUFA (polyunsaturated fatty acid); ∑C18:2 n6+ C20:2 n6+ C20:4 n6+C22:5 n6. C22:5w6.

h n-3 PUFA (polyunsaturated fatty acid); ∑C18:3 n3+ C18:4 n3+ C20:3 n3+ C20:5 n3+ C22:5 n3+

i SFA (Saturated fatty acid); ∑C2+C4+ C6+ C8+ C10+C12+C13+ C14+ C15+ C16+ C18+ C20+C22+ C24.

j MSFA; ∑C16:1 n9+ C16:1 n7+ C17:1 n9+ C18:1 n9+ C18:1 n7+ C20:1 n9+ C22:1 n11+ C22:1 n9+ ​C24:1 n9.

k UFA; ∑C16:1 n9+ C16:1 n7+ C17:1 n9+ trans vaccinic acid+ C18:1 n9+ C18:1 n7+ C18:2 n6+ C18:3 n6 + C18:3 n3+ cis-9, trans-11 CLA+ trans-10, cis-12 CLA+ C18:4 n3+ C20:1 n9+ C20:2 n6+ C20:3 n6+ C20:4 n6+ C20:3 n3+ C20:5 n3+ C22:1 n11+ C22:1 n9+ C22:5 n6+ C22:5 n3+ C24:1 n9.

**Table 3:** Methylation procedure.

Sample	Method	Reference
Blank and Milk	BF_3_/methanol	Association of official analytical chemists [7]
Blank and Milk	Methanolic HCl followed by NaOCH_3_ 0.5 N	–
Blank	NaOCH_3_/methanol (0.5 N)	Kramer et al. [4]
Blank	NaOCH_3_/methanol (1 N)	Christin [5]
Blank	Tetramethylguanidine/methanol	Shantha et al. [6]
Blank	NaOCH_3_/methanol 0.5 N followed by BF_3_/methanol	Kramer et al. [4]
Blank	NaOCH_3_/methanol 1 N followed by BF_3_/methanol	Kramer et al. [4]
Blank	Tetramethylguanidine/methanol followed by BF_3_/methanol	Tetramethylguanidine/ methanol [6] followed by BF_3_/methanol [7]
Blank	NaOCH_3_/methanol 0.5 N followed by methanolic HCl	–
Blank	NaOCH_3_/methanol 1 N followed by methanolic HCl	–
Blank	Tetramethylguanidine/methanol followed by methanolic HCl	Tetramethylguanidine/methanol [6] followed by methanolic HCl [8]

## 2 Experimental design, materials and methods

### 2.1 Extraction procedure

Lipids were extracted in a mixture of chloroform and methanol according to the method of Bligh and Dyer [1], using modification and recommendations published by Jensen [2].

### 2.2 Methylation procedure

In the present study, combination of methanolic HCl (5% acetyl chloride/methanol) and NaOCH_3_ developed as a new method. The samples (blank and milk) were acidified with 1.00 mL of methanolic HCl at 80 °C for 10 min, and then fatty acid methyl esters were extracted with 2.00 mL of pentane. After evaporation of the pentane, fatty acids were methylated with 2.00 mL of NaOCH_3_/methanol (0.5 N) for 10 min at 50 °C.

Due to the low content of C17:0 in dairy products such as cow milk, this fatty acid used as internal standard for quantification the milk fatty acid [2], [3]. To examine the effect of different methylation procedure on response of C17:0, blank samples were analysed with 11methods in anhydrous condition (Table 3). NaOCH_3_/methanol (0.5 N) was heated for 10 min at 50 °C [4]. NaOCH_3_/methanol (1 N) method was carried out according to Christin [5]. Samples were dissolved in 2.00 mL of NaOCH_3_/methanol for 5 min at room temperature. Tetramethylguanidine/methanol was heated for 2 min at 100 °C [6]. Also, several combinations of methylation procedures were tested: NaOCH_3_/methanol (0.5 and 1 N) for 10 min at 50 °C followed by an excess of BF_3_/methanol for 10 min at 50 °C [7], tetramethylguanidine/methanol for 2 min at 100 °C [6] followed by an excess addition of BF_3_/methanol for 10 min at 50 °C, tetramethylguanidine/methanol for 2 min at 100 °C [6] followed by excess addition of methanolic HCl for 10 min at 80 °C [8], NaOCH_3_/methanol (0.5 and 1 N) for 10 min at 50 °C followed by an excess addition of methanolic HCl for 10 min at 80 °C and methanolic HCl for 10 min at 80 °C followed by an excess addition of NaOCH_3_/methanol (0.5 and 1 N) for 10 min at 50 °C.

### 2.3 Gas chromatography procedure

Fatty acid methyl esters were analysed with a gas chromatograph (Hewlett-Packard 6890 series, Agilent Technologies, Palo Alto, CA, USA) equipped with an automatic column injector (Hewlet Packard 7673), a capillary column of 30 m × 0.32 mm i.d., 0.25 μm thickness (Omegawax 320; Supelco, Sigma- Aldrich), and a flame ionization detector.

### 2.4 Statistical analysis

The results from milk fatty acid were compared by using unpaired t-test, and *P*- values of <0.05 were considered significant.
